# Readout and control of an endofullerene electronic spin

**DOI:** 10.1038/s41467-020-20202-3

**Published:** 2020-12-17

**Authors:** Dinesh Pinto, Domenico Paone, Bastian Kern, Tim Dierker, René Wieczorek, Aparajita Singha, Durga Dasari, Amit Finkler, Wolfgang Harneit, Jörg Wrachtrup, Klaus Kern

**Affiliations:** 1 Max Planck Institute for Solid State Research, Stuttgart, Germany; 2 Institut de Physique, École Polytechnique Fédérale de Lausanne, Lausanne, Switzerland; 3 Physikalisches Institut, University of Stuttgart, Stuttgart, Germany; 4 Quantum Spintronics Group, University of Osnabrück, Osnabrück, Germany; 5 Department of Chemical and Biological Physics, Weizmann Institute of Science, Rehovot, Israel

**Keywords:** Carbon nanotubes and fullerenes, Magnetic properties and materials, Spintronics, Quantum metrology

## Abstract

Atomic spins for quantum technologies need to be individually addressed and positioned with nanoscale precision. C_60_ fullerene cages offer a robust packaging for atomic spins, while allowing in-situ physical positioning at the nanoscale. However, achieving single-spin level readout and control of endofullerenes has so far remained elusive. In this work, we demonstrate electron paramagnetic resonance on an encapsulated nitrogen spin (^14^N@C_60_) within a C_60_ matrix using a single near-surface nitrogen vacancy (NV) center in diamond at 4.7 K. Exploiting the strong magnetic dipolar interaction between the NV and endofullerene electronic spins, we demonstrate radio-frequency pulse controlled Rabi oscillations and measure spin-echos on an encapsulated spin. Modeling the results using second-order perturbation theory reveals an enhanced hyperfine interaction and zero-field splitting, possibly caused by surface adsorption on diamond. These results demonstrate the first step towards controlling single endofullerenes, and possibly building large-scale endofullerene quantum machines, which can be scaled using standard positioning or self-assembly methods.

## Abstract (web summary)

Encasing a single atom within a fullerene (C_60_) cage can create a robustly packaged single atomic spin system. Here, the authors perform electron paramagnetic resonance on a single encased spin using a diamond NV-center, demonstrating the first steps in controlling single spins in fullerene cages.

## Introduction

Building quantum technologies requires the constituent quantum elements to be precisely positioned and addressable^1^. Fullerene cages have demonstrated positioning with nanoscale precision^2^. The cages can be dragged into position with a scanning tunneling microscope tip^3^, or can be packed into one-dimensional arrays within carbon nanotubes (CNTs)^4^. Recent experiments have even demonstrated that C_60_ can self-assemble on monolayer graphyne sheets^5^. To address the fullerene system, an atomic spin is encapsulated within the diamagnetic enclosure of the C_60_ cage (also known as an endofullerene). The encapsulated spin is created by ion implantation, and retains its free atomic electron configuration^6^. Previous electron resonance experiments on the encapsulated spin have utilized ensemble techniques, involving  ≈10^6^ spins. However, electron paramagnetic resonance (EPR) on single endofullerenes demonstrating readout and control has remained elusive^7–9^.

**Fig. 1:**
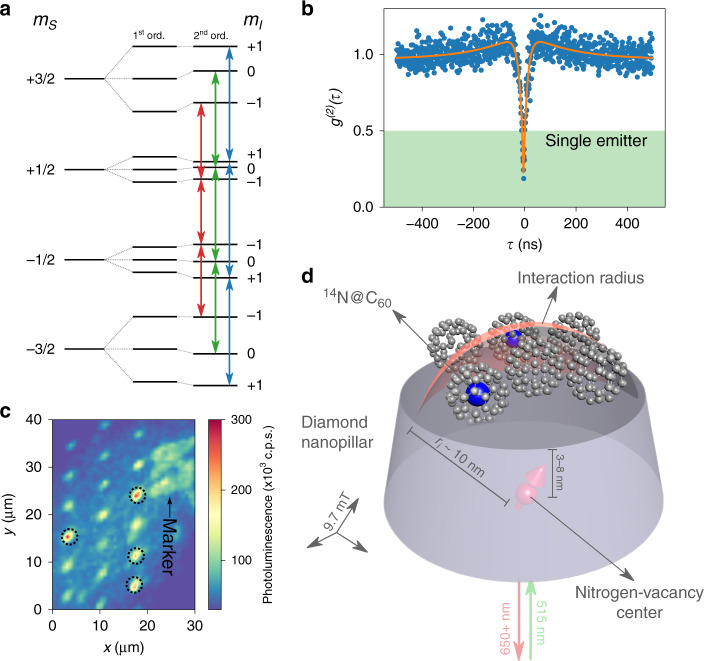
Schematic of the experiment. **a** Energy level scheme of ^14^N@C_60_ ground state (*S* = 3/2, *I* = 1) for a C_60_ cage with icosahedral symmetry and second-order corrections of order *A*^2^/(*γ*_ef_*B*) ≈ 1 MHz. Colors indicate transitions between spin states with *m*_*I*_ = −1 (red), *m*_*I*_ = 0 (green), and *m*_*I*_ = −1 (blue). **b** Single NV centers identified using Hanbury Brown–Twiss photon autocorrelation with *g*^2^(0) < 0.5 (shaded region). **c** Confocal image of red fluorescence from N@C_60_-coated diamond nanopillar arrays showing several luminescent single NV centers (dotted circles) at cryogenic conditions. The diamond surface is labeled with markers to probe the same NV site at different experimental conditions. **d** Schematic of a single near-surface NV center with a disordered network of empty and filled C_60_ cages within its interaction radius.

In this work, we focus on the endofullerene N@C_60_. N@C_60_ is formed by a single ^14^N atom sitting at the centrosymmetric *I*_*h*_ position of a C_60_ cage. The nitrogen atom retains its single-particle eigenvalues, in particular its electronic ground-state quartet^10,11^. The ground state is formed by an electronic spin *S* = 3/2 system residing in the hyperfine field of a spin *I* = 1 nucleus. The experiment is performed at low magnetic fields (*B* ≈ 10 mT), leading to second-order corrections to the N@C_60_ energy-level scheme of order *A*^2^/(*γ*_ef_*B*) ≈ 1 MHz, where *A* is the N@C_60_ hyperfine constant and *γ*_*e*_*B* is the electronic Zeeman shift. This is shown in Fig. 1a. We utilize the dipolar interaction of the N@C_60_ electronic spin with a local readout sensor to demonstrate single-spin level endofullerene spin readout and control.

## Results

### Sensor and system

For the local readout sensor, we use single near-surface (3–8 nm deep) nitrogen vacancy (NV) centers in diamond^12^. The NV center is ubiquitous as a quantum sensor whose robust solid-state packaging and unique energy level structure make it ideal as a noninvasive local magnetic field probe^13,14^. Briefly, it consists of a substitutional nitrogen atom neighboring a vacancy site in the diamond lattice, creating a triplet ground state that can be polarized and readout optically. Single NV centers were isolated using Hanbury Brown–Twiss measurements (Fig. 1b, see Supplementary Section 1.3). The single NV centers were located using standard confocal microscopy and are labeled to probe the same NV site at different experimental conditions (Fig. 1c). The optical collection efficiency of the NV centers was enhanced by guiding the emitted light using tapered nanowaveguides structured directly onto the diamond substrate^15^.

The diamond surface was cleaned and oxygen-terminated by tri-acid boiling (see “Methods”). After cleaning, a disordered layer of empty (C_60_) and filled (N@C_60_) cages was created by drop-casting and air-drying N@C_60_ solution on the surface. The filling factor of the cages (1.0(1) × 10^−4^) and concentration in solution (1 μLL^−1^) was tuned for single N@C_60_ spin detection within the NV center sensing radius of  ≈10 nm. From the filling factor and concentration, we calculate a probability of ≈4.5% of coupling to a single N@C_60_ spin (see Supplementary Section 1.4). In other words, on average only 1 in 22 NV centers will couple to an N@C_60_ spin. The probability of coupling to more than a single spin falls rapidly, and is ≈0.2% for coupling to two N@C_60_ spins. However, we note that the statistical fluctuations in these values can be large for single-spin coupling.

### Dipolar interaction model

Denoting the interacting spins as *S*_nv_ (NV electronic spin), *S*_ef_ (endofullerene electronic spin), and *I*_ef_ (endofullerene nuclear spin), and applying a static magnetic field ${{\bf{B}}}_{0}={B}_{0}\hat{z}$ aligned to the NV quantization axis, the spin Hamiltonian (in units of *h*) of the system reads as:

1 $$\hat{{\mathcal{H}}}={\hat{{\mathcal{H}}}}_{{\rm{nv}}}+\underbrace{{{\gamma }_{{\rm{ef}}}{\hat{B}}_{0}{\hat{S}}_{{\rm{ef}}}+A{\hat{S}}_{{\rm{ef}}}{\hat{I}}_{{\rm{ef}}}+D{\hat{S}}_{{\rm{ef}}}{\hat{S}}_{{\rm{ef}}}}} _{{\hat{\mathcal{H}}}_{\rm{ef}}} + \underbrace{{J{\hat{S}}_{{\rm{nv}}}{\hat{S}}_{\rm{ef}}}} _{{{\hat{\mathcal{H}}}_{{\rm{nv}},{\rm{ef}}}}}.$$

Here, *γ*_ef_ = *gμ*_*B*_/*h* ≈ 28 GHz/T is the gyromagnetic ratio of the endofullerene electronic spin, ∣*A*∣ ≈ 15.87 MHz is the isotropic hyperfine coupling of the encapsulated ^14^N, and ∣*D*∣ is the zero-field interaction strength appearing in the absence of perfect icosahedral cage symmetry^16^. The interaction Hamiltonian ${{\mathcal{H}}}_{{\rm{nv}},{\rm{ef}}}$ assumes higher external fields *B*_0_ relative to the hyperfine interaction *A* and dipole–dipole interactions *J*, i.e., *γ*_ef_*B*_0_ ≫ *A* ≫ *J*. The dipole–dipole coupling strength ∣*J*∣ = *μ*_0_*ℏγ*_nv_*γ*_ef_/(2*r*^3^) ≈ 52 MHz/(*r*/nm)^3^ varies with NV and endofullerene separation *r* (in nm)^17^.

### Single-spin EPR

**Fig. 2:**
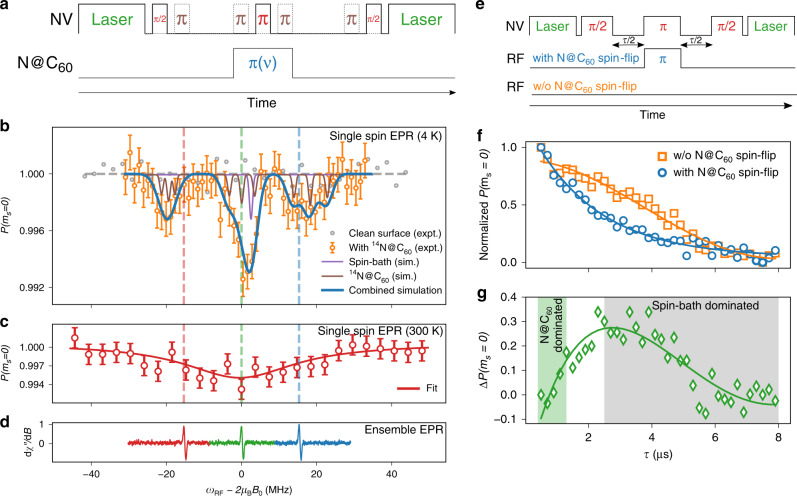
Electron spin resonance of endofullerenes. **a** Single-spin electron paramagnetic resonance (EPR) realized using double electron–electron resonance (DEER) in the double-quantum (DQ) basis. Brown pulses (dotted lines) and red pulses (continuous lines) address 0 → +1 and 0 → −1 nitrogen vacancy (NV) transitions, respectively. **b** Low-temperature DEER-DQ spectroscopy on a single NV center in diamond. The solid brown and purple lines indicate the simulated spectrum of a single N@C_60_, and weakly coupled spin-bath (*g* = 2.03), respectively (linewidths reduced to 1 MHz for clarity). The solid blue line indicates the combined simulation with experimental linewidth. The simulated endofullerene has an isotropic hyperfine constant *a* = 19 MHz, axial zero-field splitting *D* = 1.52 MHz and a static magnetic field *B*_0_ = 9.697 mT (see Supplementary Section 2). Dotted vertical lines indicate positions of ensemble EPR hyperfine components. Error bars depict photon shot noise. **c** Spectrum decays under room-temperature (RT) thermo-optical load, solid line indicates fit to single Lorentzian model. Dotted lines indicate positions of ensemble EPR hyperfine components. Error bars depict photon shot noise. **d** Ensemble solution EPR data on the same N@C_60_ sample measured across ∼10^9^ spins. **e** Pulse sequence comparing NV center Hahn-echo decoherence in presence (first RF sequence) and absence (second RF sequence) of an RF spin-flip on N@C_60_. **f** Direct comparison of the temporal evolution of the NV Hahn-echo signal with and without a spin-flip on the coupled N@C_60_. **g** Difference in NV center decoherence at short evolution times versus longer evolution times reveals the clear separation of timescales at which the NV–N@C_60_ interaction is dominant.

The dipole–dipole interaction was probed at several single NV centers using pulsed electron–electron double resonance (PELDOR or DEER) spectroscopy^18,19^. DEER spectroscopy utilizes periodic microwave (MW) pulses in a spin-echo sequence to preserve the phase coherence and enhance the magnetic field sensitivity of the NV center^20,21^. The central spin-flip of the NV center is synchronized with a radio-frequency (RF) spin-flip pulse in a lock-in scheme. When the frequency of the RF pulse matches the transition frequency of an external spin, the NV center basis states pick up a relative phase proportional to the magnetic field of the external spin. To increase the net phase pickup and suppress common mode noise, we chose the *m*_*s*_ = {±1} NV electronic spin sublevels as sensor basis states^18^. This double-quantum (DQ) pulse scheme is shown in Fig. 2a.

 Figure 2b shows the single-spin EPR spectrum of N@C_60_ at 4.7 K under an external magnetic field of 9.697 mT. The spectrum is obtained on averaging many individual single-shot experiments, and therefore represents a temporal average in which the N@C_60_ spin system assumes all allowed configurations with a probability given by the temperature, and possibly by saturation effects due to finite shot repetition times. Three resonance peaks at 251.5(5), 271.4(1), and 288.7(7) MHz mark the hyperfine splitting of the endofullerene nitrogen. Note that after cleaning the diamond surface, the signal disappeared completely, indicating that the signal stems from the surface and not from impurities within the diamond itself.

Comparing single-endofullerene EPR (Fig. 2b) with ensemble measurements (Fig. 2d) on the same sample reveals good agreement between the central and upper hyperfine components. However, the single-endofullerene EPR spectrum shows two additional features—(i) the lowest-energy hyperfine transition is shifted downward by  ≈4 MHz, and (ii) the EPR linewidths are broadened. Both of these experimental features can be modeled using second-order perturbation theory (see Supplementary Section 2), and the result of the simulation is shown in Fig. 2b as a solid blue line. Firstly, the position of the three peaks can be best reproduced by using an isotropic hyperfine constant of 19 MHz for the spin-3/2 species instead of the usual 15.85 MHz, and an axial zero-field splitting (parameterized as *D* in Eq. (1)) of 1.52 MHz. The former shifts the frequency of the hyperfine satellites relative to the central peak and the latter leads to an additional splitting within each hyperfine line. The apparent enhancement of these parameters may be due to either covalent^22–24^, or non-covalent^25^ chemical interactions between the fullerene and the diamond surface. Secondly, the simulation uses an overall line broadening of 4 MHz, which is in agreement with the typical experimental power broadening expected from Fourier transformed 100 ns RF pulses 1/(*π* × 100 ns) ≈ 3.3 MHz. The peak intensities are modeled considering a weak coupling to a spin-bath (*g* = 2.03), which may originate from electronic spins in the drop-coated solution^26,27^. Another possible interpretation of the single-endofullerene EPR peak intensities can be made by invoking nonthermal spin distributions, potentially caused by the NV intersystem crossings during laser excitation (see Supplementary Section 2.4).

At 4.7 K, we could observe the single-spin EPR signal over weeks, and N@C_60_ itself is known to be thermally stable up to 550 K. However, when placing N@C_60_ under the exposure of strong laser irradiation, ensemble experiments have demonstrated that it can undergo thermo-optical decay at temperatures well <550 K (ref. ^28^). To test this, we raised the temperature of the system to 300 K and repeated the DEER measurement. We observe a complete suppression of any discernible side peaks along with the formation of a broad featureless central dip (Fig. 2c). This behavior is consistent with previous attempts at room temperature endofullerene spin readout under laser irradiation^8^. Ensemble EPR measurements have attributed the decay mechanism to the formation of a radical species in an adiabatic photoreaction^29^. Another possibility is the thermally assisted rapid dephasing of an intact N@C_60_ spin.

### N@C_60_ spin relaxation and control

The sensor relaxation times can provide crucial information about the environment, such as the distance to a dipolar-coupled spin. To determine the distance of the endofullerene from the NV center, we make a direct comparison of the temporal evolution of the NV phase pickup with and without a spin-flip on the central N@C_60_ spin transition (Fig. 2f and see Supplementary Section 1.5). The spin-echo sequence on the NV center shown in Fig. 2e mainly refocuses the phase imprinted by nearby spins (i.e., a strongly coupled spin-bath), and the simple decoupling sequence does not affect the dephasing caused by farther spins (i.e., a weakly coupled spin-bath). Hence the long-time behavior is unaffected by the *π*-pulse. As we start to flip the N@C_60_ spin, depending on the timescale of the phase evolution, the NV center either dominantly dephases due to its coupling to the N@C_60_, or to the weakly coupled spin-bath. This behavior is seen in Fig. 2g, where a strong initial dephasing due to the NV–N@C_60_ interaction slowly relaxes to the dephasing caused by the spin-bath. Hence, the difference between the two dephasing curves should display the expected behavior, i.e., a transition from a faster dephasing caused by external spins toward a slower dephasing from the local spin-bath. Using a simple rate equation model (for full analysis see Supplementary Section 1.5 and ref. ^19^), we extract an NV–N@C_60_ coupling strength of ≃0.29(2) MHz from the initial linear dephasing, corresponding to an NV–N@C_60_ separation ≃5.6(1) nm. Considering the possibility of the additional presence of a finite number (<10) of unknown bath spins, the uncertainty in distance measurements is slightly affected with an additional error bar of  ±1 nm.

**Fig. 3:**
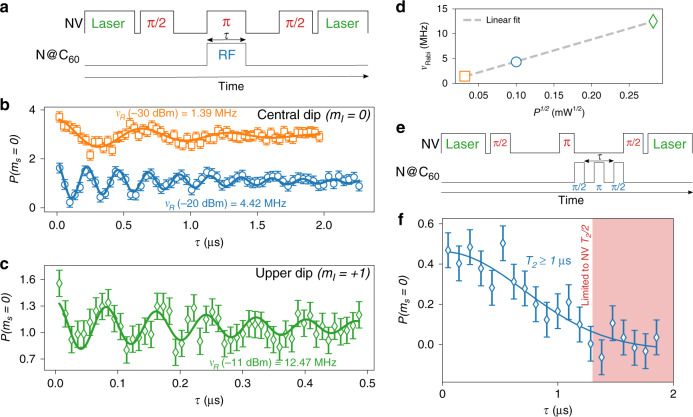
Relaxation and coherence of an endofullerene spin. **a** Pulse scheme for driving Rabi oscillations on endofullerene spins and probing with a single nitrogen vacancy (NV) center . **b**, **c** Rabi oscillations on the endofullerene central (*m*_*I*_ = 0) and upper (*m*_*I*_ = +1) hyperfine components. Solid line is fit to an exponentially decaying sinusoid. Error bars indicate photon shot noise. **d** Characteristic square-root behavior of Rabi frequency with RF driving power. **e** Pulse scheme for probing endofullerene spin-echo phase coherence. **f** Spin-echo measurements place a lower limit of *T*_2_ ≥ 1 μs on the phase coherence times. Solid line indicates exponentially decaying sinusoidal fit, error bars indicate photon shot noise.

The dephasing and relaxation times of N@C_60_ were long enough to generate and measure spin coherence, as shown by the Rabi oscillations (pulse sequence Fig. 3a). The Rabi nutation of the endofullerene spin is demonstrated in Fig. 3b, c. Rabi oscillations are recorded by fixing the RF frequency at the central (*m*_*I*_ = 0) and upper (*m*_*I*_ = +1) peaks in Fig. 2b, and varying the pulse duration time *τ*. The Rabi frequency displays the characteristic square-root scaling with driving power (Fig. 3d). The Rabi control allows for rapid pulsed control of the endofullerene spin-level populations, with spin-state switching rates tunable up to *ν*_*R*_ = 12.47(1) MHz.

Performing more general control sequences demands phase coherence (T_2_). To measure this, we performed a spin echo on the endofullerene within half of the free evolution time in the NV spin echo (Fig. 3e). The result is a characteristic Hahn decay in Fig. 3f, with an observed decay time of T_2_ ≥ 1 μs. For NV centers with a spin-echo coherence of ${T}_{2}^{{\mathrm{NV}}}\approx 2.5\,\upmu {\rm{s}}$, this leads to a maximum measurable coherence time of ${T}_{2}^{{\mathrm{NV}}}/2\approx 1.25\,\upmu {\rm{s}}$. Thus, the time represents a lower limit on N@C_60_ coherence due to the measurement being constrained to a single branch of the NV spin echo. The maximum measured ensemble endofullerene coherence time is ~190 μs (ref. ^29^), and thus probing and controlling such spins requires near-surface NV centers with significantly longer coherence times. This can be achieved through the chemical surface modification of diamond, or by doping the diamond with n-type impurities^30^.

## Discussion

In conclusion, we have demonstrated electron spin resonance with readout and control on the endofullerene ^14^N @C_60_, using a near-surface nitrogen vacancy center in diamond. The surface adsorption on to the diamond surface appears to enhance isotropic hyperfine constants and zero-field splitting of the fullerene cage. The use of fullerenes, such as ^31^P@C_60_, which has hyperfine interactions an order of magnitude larger than ^14^N@C_60_, may permit spin control without strong surface effects. The current results represent the first steps toward controlling single endofullerenes and combining our result with recent advances in C_60_ self-assembly, or fullerene packed CNTs, opens a path for scaling endofullerene systems^5^. Furthermore, the nuclear spin of the encapsulated atom allows for nuclear quantum memories interfaced with electron spin “buses”^31,32^. This can be realized with pulse schemes, such as Hartman-Hahn, which transfer polarization from the NV center to the endofullerene electronic and nuclear spins (see Supplementary Section 3). Combining these techniques together open up incredibly exciting possibilities of building large-scale endofullerene quantum machines^1^.

## Methods

### Experimental setup

The single-spin EPR measurements on N@C_60_-coated diamond samples were performed with a home-built low-temperature (4.7 K) and ultrahigh vacuum (10^−10^ mbar) setup capable of confocal microscopy and pulsed MW/RF control^21,33^. The ensemble EPR measurements were performed on an X-band cw-EPR spectrometer (see Supplementary Section 1.1).

### Diamond preparation and characterization

All experiments were performed on a 30 μm thick electronic grade [100] diamond made, using chemical vapor deposition by Element Six (UK) Ltd. The diamond was implanted with ^15^N at an energy of 5 keV and subsequently annealed at 975 ^∘^C for 2 h. Nanopillar waveguides (base diameter 700 nm, tip diameter 400 nm, and height 1 μm) were etched into the diamond to increase optical collection efficiency. The diamond surface was cleaned and oxygen terminated by boiling in a tri-acid mixture (1:1:1, HNO_3_:H_2_SO_4_:HClO_4_) at 200 ^∘^C for 5 h.

### ^14^N@C_60_ preparation and characterization

A powder sample of ^14^N@C_60_ with a filling factor of 10^−4^ was dissolved in toluene to a concentration of 0.1 μLL^−1^ using ultrasonic dissolution. A total of 1 μL of this solution was drop-coated on the diamond surface under ambient conditions.

### NV spin initialization, readout, and control

NV spins were initialized and readout by a 515 nm laser gated by two acousto-optic modulators in a double-pass configuration. The laser was focused using a low-temperature objective (NA = 0.82). The red light emitted by the NV center was long-pass filtered at 650 nm, and monitored by two crossed avalanche photodiodes in a HBT configuration. Spin control was achieved by MW and RF pulses through a gold wire (thickness 20 μm) fabricated across the diamond surface. Pulses were generated by local oscillators multiplied with TTL signals from a pulse generator, power combined, and amplified (40 dB gain). DQ sequences used separate MW sources to address the 0 → ±1 transitions in a two-pulse scheme^20^.

### Theoretical model and numerical simulation methods

The magnetic field at NV center was calculated using the ODMR spectrum and NV Hamiltonian. The DEER spectrum was calculated using second-order perturbation theory, assuming that the unperturbed Hamiltonian is represented by the electron Zeeman interaction, and the perturbation by the the hyperfine and zero-field interactions.

## Supplementary information

Supplementary Information

## Data Availability

The data that support the findings of this study are available from the corresponding author on reasonable request.
