# Supplementary material for: Readout and control of an endofullerene electronic spin
Source: Nat Commun. 2020 Dec 17;11:6405. doi: 10.1038/s41467-020-20202-3 (PMC7746685; doi:10.1038/s41467-020-20202-3)
Supplement: Supplementary file 1 — Supplementary Information [file 41467_2020_20202_MOESM1_ESM.pdf]

Supplementary Information

Readout and control of an endofullerene electronic spin

Dinesh Pinto[1,2], Domenico Paone[1,3], Bastian Kern[1], Tim Dierker[4], René Wieczorek[4], Aparajita Singha[1], Durga Dasari[3], Amit Finkler[3,5], Wolfgang Harneit[4], Jörg Wrachtrup[1,3], and Klaus Kern[*1,2]

[1]Max Planck Institute for Solid State Research, Stuttgart, Germany
[2]Institut de Physique, École Polytechnique Fédérale de Lausanne, Lausanne, Switzerland
[3]3. Physikalisches Institut, University of Stuttgart, Stuttgart, Germany
[4]Quantum Spintronics Group, University of Osnabrück, Osnabrück, Germany
[5]Department of Chemical and Biological Physics, Weizmann Institute of Science, Rehovot, Israel

Contents

1 Probing single N@C_{60} spins **2**
1.1 Preparation and purification of N@C_{60} 2
1.2 Creating a disordered matrix of N@C_{60} on diamond 2
1.3 Single near-surface NV centers 3
1.4 NV coupling to a single N@C_{60} 4
1.5 NV-N@C_{60} coupling strength and distance 5
1.6 Experimental setup 7

2 CW Spectrum according to second order perturbation theory **9**
2.1 Dependence on zero-field axis 13
2.2 Including anisotropic hyperfine interaction 15
2.3 Enhanced isotropic hyperfine and axial zero-field interaction 16
2.4 Alternative interpretation of peak intensity: Non-thermal spin distributions. 17

3 Scalable endofullerene quantum structures **19**

*e-mail:k.kern@fkf.mpg.de

1 Probing single N@C$_{60}$ spins

1.1 Preparation and purification of N@C$_{60}$

The N@C$_{60}$ sample was produced by the low-energy implantation of ^{14}N inside C$_{60}$ [1]. To summarize, an effusion cell was used to sublimate a thin C$_{60}$/C$_{70}$ film (mass content approx. 80:20) on a LN$_2$ cooled copper target corresponding to approximately 0.5 g crude material. During the formation of this film, the copper target was bombarded by 0.1 keV ^{14}N and ^{14}N$_2$ ions from an ion source. A fraction of these ions enter the fullerene cages to form N@C$_{60}$ and N@C$_{70}$. This implantation process yields a filling factor (N@C$_{60}$/[C$_{60}$ + N@C$_{60}$]) of around 5×10^{-4}. To separate C$_{60}$/N@C$_{60}$ from C$_{70}$/N@C$_{70}$, higher fullerenes, and non-fullerene by-products, purification by filtering and plug-filtration [2] was conducted. Eventually, enrichment of N@C$_{60}$ towards C$_{60}$ was accomplished by high pressure liquid chromatography (HPLC). This method capitalizes on the small difference in polarizability of the two species to separate them via molecular interactions with a pyrene-functionalized column bed (COSMOSIL Buckyprep, Nacalai Inc.).

The C$_{60}$/N@C$_{60}$ content was characterized by an analytical HPLC protocol utilizing an UV detector which was calibrated against a C$_{60}$ standard. The amount of N@C$_{60}$ was determined using X band cw-EPR spectroscopy (microwave power: 10 µW; modulation amplitude: 0.01 mT; resolution: $5 \cdot 10^{-4}$ mT/bin; sweep rate: 0.02 mT/s.). Spin counting was enabled by TEMPO calibration. Analysis of the N@C$_{60}$ sample yields a purity of 99.5 % with C$_{120}$ as main impurity, a filling factor of $1.0(0.1) \cdot 10-4$ and a hyperfine coupling strength of 15.8(1) MHz.

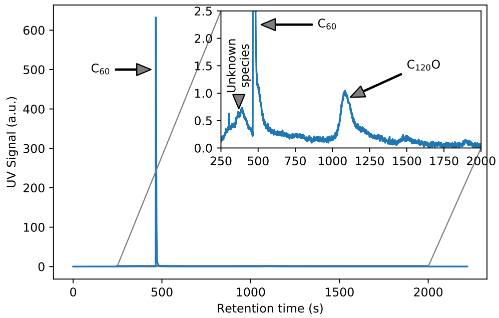

a

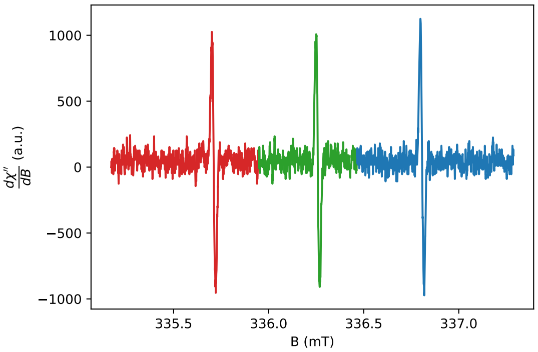

b

Figure S1: **N@C$_{60}$ purification and characterization.** **a,** Chromatogram of N@C$_{60}$. **b,** X-band cw-EPR on N@C$_{60}$, ff=10^{-4}

1.2 Creating a disordered matrix of N@C$_{60}$ on diamond

The purified N@C$_{60}$ powder (21.8 mg, filling factor 10^{-4}) was dissolved in 30 mL of toluene to a concentration of 0.1 µL L^{-1} under ambient conditions. The solution was then placed in an ultrasonic bath for a period of 15 min. After complete dissolution, 1 µL of the solution was drop-cast on the surface of diamond and air-dried under ambient conditions. The diamond was then transferred to an ultra-high vacuum (10^{-10} mbar) chamber via a loadlock. Within the UHV chamber the sample was thermally anchored to the base of a liquid ^{4}He bath cryostat (4.2 K). The experimental setup is described briefly in subsection 1.6.

1.3 Single near-surface NV centers

The diamond itself was an electronic grade CVD [100] diamond (from Element Six) of thickness 30 µm. The diamond was implanted with ^{15}N at an energy of 5 keV and subsequently annealed at 975°C for 2 h. Nanopillar waveguides (base diameter 700 nm, tip diameter 400 nm, height 1 µm) were etched into the diamond to increase the optical collection efficiency [3]. The diamond surface was cleaned and oxygen terminated by boiling in a three acid mixture ($1:1:1, HNO_3 : H_2SO_4 : HClO_4$) at 200°C for 5 h. The depth of the implanted NV centers is 3 nm to 8 nm. The diamond was imaged using a confocal microscope (Figure S3). Single NV sites were isolated using Hanbury-Brown-Twiss measurements with a zero-delay autocorrelation dip of under 0.5 (Figure S2a). The spin-state calculation was performed by comparing bright and dark state contrast during the laser readout pulse (Figure S2b). ODMR and Rabi oscillations were performed on every NV site to measure field strength and readout contrast (Figure S2c). In the measurement, 30 NV centers were probed, out of which a single NV showed coupling to N@C_{60}, indicating that the concentration in solution was likely lower than previously calculated (subsection 1.2). The NV centers probed during DQT measurements were excited at a frequency of 2.605 81 GHz and 3.149 50 GHz, for the $0 \to -1$ and $0 \to +1$ transitions respectively.

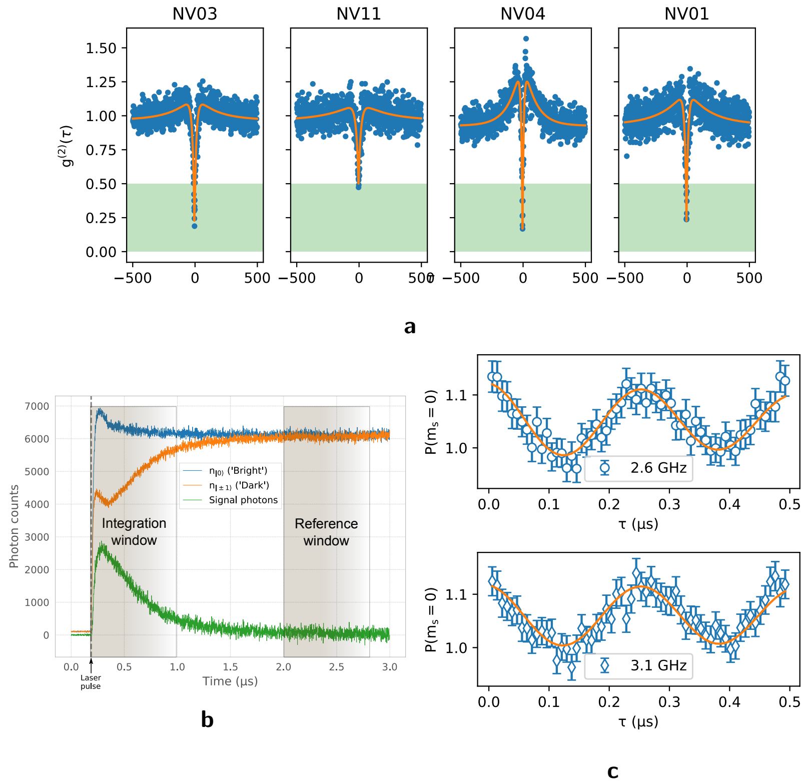

Figure S2: **Single NV center properties.** **a**, $g^{(2)}$-correlation measurements from a sub-sample of probed NV centers. **b**, Comparison of bright and dark state contrast for NV03 with integration and reference windows used for spin-state calculation. **c**, Rabi oscillations on upper and lower ODMR transitions of NV03.

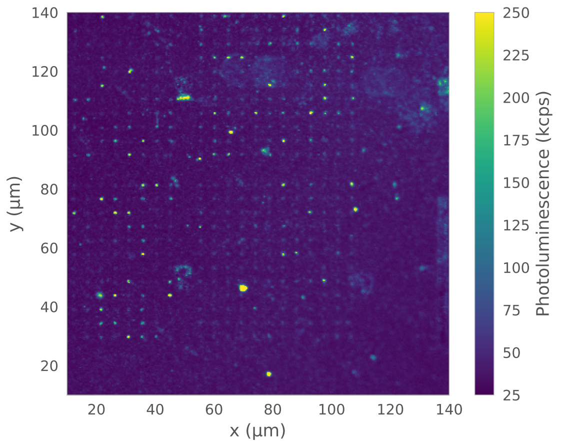

Figure S3: **Diamond surface.** Confocal image of diamond surface at ambient conditions.

1.4 NV coupling to a single N@C_{60}

Using the NV depth extracted from subsection 1.5, NV T_2 from Hahn-Echo measurements, filling factor from subsection 1.1 and taking the C_{60} cage radius from Ref. [4], we obtain the following:

- NV depth $\approx$ 5 nm
- NV interaction radius (extracted from T_2 time) $\approx$ 10 nm
- Filling factor (N@C_{60}/C_{60}) $\approx 10^{-4}$
- Radius of C_{60} cage $\approx$ 0.7 nm

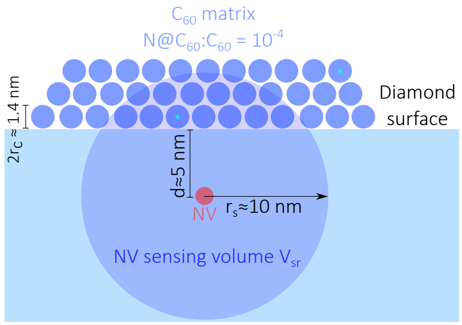

Figure S4: **NV coupling to a single N@C_{60}.** Schematic diagram depicting the interaction radius of the NV center (large purple circle, volume V_{sr}) and fullerene cages on diamond surface (small purple circles, volume $V_{C_{60}}$). Some fullerene cages ($\approx$1 in 10^4), will have a ^{14}N spin inside it (cyan dot).

Assuming a maximally dense packing of C_{60} cages, we can calculate the the number of C_{60} cages within the interaction radius of a single NV center. $V_{s.r.}$ is the sensing volume of the NV center at depth d with sensing radius r_s above the surface of diamond and $V_{C_{60}}$ is the volume of a single C_{60} cage with radius r_c. The resultant number of C_{60} spins can be combined with the filling factor of $= 10^{-4}$ to extract the probability of coupling to a single N@C_{60} spin. This is depicted in Figure S4.

$$\begin{aligned}\frac{V_{s.r.}^{NV}}{V_{C_{60}}} &= \frac{\frac{\pi}{3}(r_s-d)^2(2r_s+d)}{\frac{4}{3}\pi r_c^3}\\ &= \frac{(5\ nm)^2[2(10\ nm)+5\ nm]}{4(0.7\ nm)^3}\\ &\approx \frac{654.5\ nm^3}{1.43\ nm^3} \leq 455\ \mathrm{C_{60}}\ \text{cages in NV sensing radius}\\ &\implies 455\times 10^{-4} \approx 0.045\ \mathrm{N@C_{60}}\ \text{cages in NV sensing radius}\end{aligned}$$

Thus the calculated probability of coupling to a single N@C_{60} is approximately 4.5% (or 1 in 22 centers). The probability of coupling to more than one N@C_{60} spin is much smaller, e.g. for two spins around 0.2%. However, we would like to emphasize that since we only observe one coupling the statistical fluctuation can be high. We also note that this value is susceptible to local variations in N@C_{60} density, and thus requires the solution to be uniform with N@C_{60} density. The solution used in the measurement involved ultrasonic dissolution of N@C_{60} powder in toluene solution, which is a common method for achieving uniformly dense solutions.

1.5 NV-N@C_{60} coupling strength and distance

Our measurement scheme is similar to the more generalized spin-echo double resonance (SEDOR) proposed in [5], to measure the coupling of each of the bath spin groups to the NV center. Within this scheme the dephasing of the NV spin induced by one particular group of bath spins can be probed, while the effect of all other dephasing channels (including other spin bath groups) is refocused. This measurement sequence has been used to quantify the contribution from the nuclear and electron (P1 centers) spin-baths to the total dephasing of the NV spin [5, 6] and the external spins [7]. In the employed DEER pulse-sequence we refocus only the electron spins, which could include both the known spins from the N@C_{60} and any unknown electron spin-bath. Thus, the π-pulse on the electronic spin-bath also refocuses the static noise experienced by these external spins caused by their local environments, thereby having a two-fold effect on NV spin decoherence. The NV spin-echo signal displays a Gaussian envelope decay with a decoherence time of $T_2 \simeq 4.3\,\mu\text{s}$ when all the decoherence channels are refocused. On the other hand, when some of the dephasing channels are refocused, we find the decay is well-fitted for Equation 1. The fits for the data are shown in Figure S5.

$$P \approx \exp\left(\frac{-t}{\tau}\right)\cos\left(\frac{gt}{2}\right) \tag{1}$$

where $\tau = 3.17\,\mu\text{s}$ is the coherence decay of the NV-spin determined by the magnetic environment that is not refocused due to the π-pulse performed at the frequency $\omega = 272\,\text{MHz}$. Further, this decay is modulated by the dominant coherent coupling of the external spin at the coupling rate g. A simple and well used model to explain such a behavior is determined by the Hamiltonian evolution and the decoherence of the NV spin. For the Hamiltonian we consider the dominant contribution of the dipolar interaction along the field direction (z), given by,

$$\mathcal{H} = S^z\left(\frac{g}{2}\sigma_L^z + \sum_k \frac{F_k}{2}\sigma_k^z\right) \tag{2}$$

where σ's are Pauli matrices. Here g is coupling to the target electron spin of the spin-label, and F_k are the couplings to the an unknown electron spin-bath. Further, we switch on a Markovian

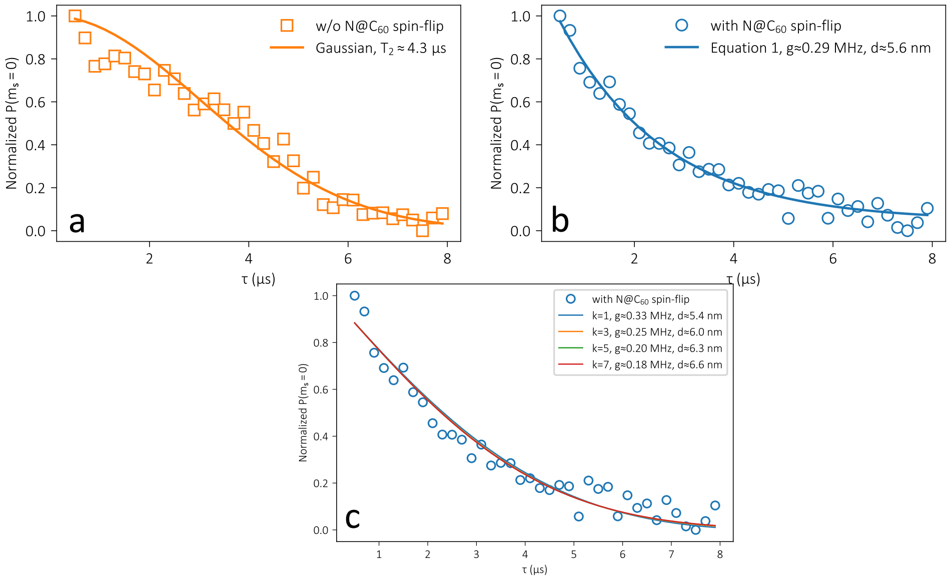

Figure S5: **Temporal evolution comparison. a,** Evolution of NV phase pickup without a spin-flip on coupled N@C_{60}. Data fit to a Gaussian decay reveals a coherence time $T_2 \simeq 4.3\,\mu\text{s}$. **b,** Evolution of NV phase pickup with a spin-flip on coupled N@C_{60}. Fitting to Equation 1 reveals coupling strength $g \simeq 0.29\,\text{MHz}$, corresponding to a separation $d \simeq 5.6\,\text{nm}$. **c,** Evolution of NV phase pickup with a spin-flip on coupled N@C_{60} and the extreme case of a few electron spins in the sensing volume. While the deviations in the distance can increase with the bath-size, this would also lead to a substantial broadening ($\gg 4$ MHz) of the DEER spectrum, which is not the case for the measurement. Thus the maximum deviation in coupling strength and distance values is within the quoted error margins. The data is fit to a multi-cosine product from Equation 3.

decoherence channel for the NV spin due to its magnetic environment, characterized by the decay constant $1/\tau$. In the case when $F_k = 0$, solving the rate equation with the characteristic decay and coherent coupling to a single electron spin with coupling g, we arrive at the same fit formula obtained above.

The best fit for the data indicates that there is a slight discrepancy between the decay constants obtained with and without the π-pulse i.e., $\tau \neq T_2$. Using $\tau = T_2$ immediately yields a high coupling value for $g \simeq 0.4\,\text{MHz}$ instead of $g \simeq 0.28\,\text{MHz}$. This could immediately hint towards the presence of additional electron spin-bath (third group), and whose effect was not refocused during the DEER pulse sequence. Including these additional electron spins and re-deriving the relation for the DEER signal [8] we obtain Equation 3.

$$P = \exp\left(\frac{-t}{\tau}\right)\cos\left(\frac{gt}{2}\right)\Pi_k \cos\left(\frac{F_k}{2}\right) \tag{3}$$

where F_k are the coupling of the NV to additional electron spins. In including these third group of electron spins with couplings F_k to explain this discrepancy, we find ambiguities as a large number of possible combinations of the values of F_k could find a better fit for the decay of DEER signal. Thus, estimating the unknown number in the additional electronic spin-bath and their coupling distributions is beyond the scope of the current work. We instead give here a qualitative error in estimating the coupling strength from the simple fit-formula, given above which is for example

including upto four additional spins would increase our uncertainty in the predicting the coupling as $0.22\,\mathrm{MHz} < g < 0.40\,\mathrm{MHz}$. As the fitted coupling values to these additional spins are in the same order one could assume the presence of an unknown electronic spin-bath close to the surface that also accounts for the observed asymmetry in the DEER spectrum. As the statistical probability of more than one N@C_{60} spin within the sensing volume is extremely low ($\approx 0.2\%$), we find that including a single or a few electron spins do not contradict our distance measurements obtained in the ideal case. We also note that any direct dipolar coupling between the N@C_{60} electron spin and electron spin-bath was neglected. Including them would lead an enhancement in the $T_{1\rho}$ of the spins, i.e., an additional equilibration channel for the target spin. As all of them are default in a fully-mixed state, and the Hamiltonian dynamics does not lead to any polarization, these dissipative channels are less effective.

1.6 Experimental setup

Schematics of the 4K-UHV setup used to perform the measurements are shown in Figure S6. The experimental setup [9] uses confocal microscopy to identify NV centers, pulsed EPR to control spins, and $g^{(2)}$-correlation to isolate single photon emitters. The NV centers are optically polarized and readout using a 515 nm laser and crossed avalanche photo-diodes (APD). Spin state control is achieved using multiple local oscillators gated by nanosecond pulses. The diamond is kept in a LT (4.2 K) and UHV (10^{-10} mbar) environment. Piezo positioners allow for diamond positioning with nanometer precision. A 3D vector magnet can generate uniform magnetic fields up to 20 mT [8].

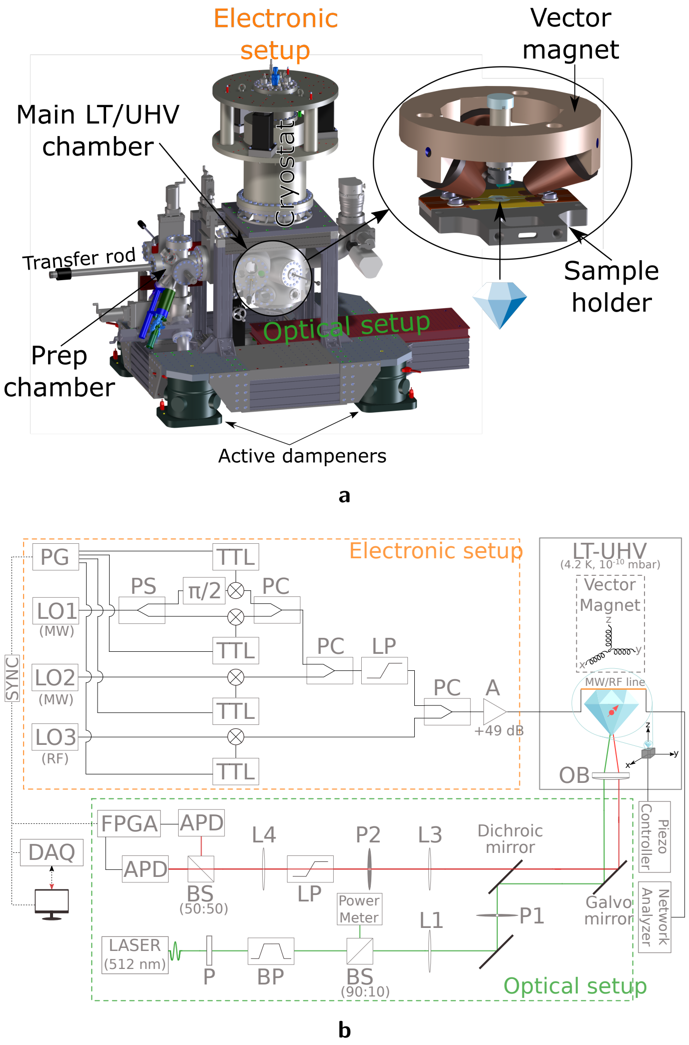

Figure S6: **Experimental setup. a**, 3D render of 4K-UHV experimental setup. **b**, Electronic circuit for double electron-electron resonance (DEER) double-quantum (DQ) spectroscopy and optical layout for confocal microscopy and photon correlation.

2 CW Spectrum according to second order perturbation theory

^{14}N trapped in a neutral C_{60} molecule

electron spin: $S = 3/2$

nuclear spin: $I = 1$

external magnetic field: $\hat{B} = \begin{pmatrix} B_x \\ B_y \\ B_z \end{pmatrix} = \begin{pmatrix} 0 \text{ mT} \\ 0 \text{ mT} \\ 9.697 \text{ mT} \end{pmatrix}$

N@C_{60} Hamiltonian:

$$\begin{aligned} \frac{\mathcal{H}}{h} &= \frac{H_{EZ}}{h} && + \frac{H_{ZF}}{h} && + \frac{H_{HF}}{h} \\ &= \frac{\mu_B}{h}\,\hat{B}^T\,\hat{g}\,\hat{S} && + \hat{S}^T\,\hat{D}\,\hat{S} && + \hat{S}^T\,\hat{A}\,\hat{I} \\ &= \frac{g\mu_B}{h}\left(B_x\hat{S}_x + B_y\hat{S}_y + B_z\hat{S}_z\right) && + \frac{D}{3}\left(2\hat{S}_z^2 - \hat{S}_y^2 - \hat{S}_x^2\right) && + a\left(\hat{S}_x\,\hat{I}_x + \hat{S}_y\,\hat{I}_y + \hat{S}_z\,\hat{I}_z\right) \end{aligned}$$

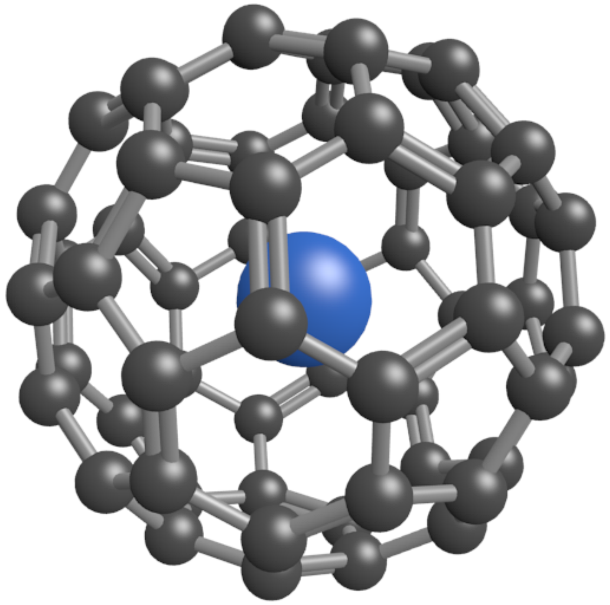

When considering the above stated Hamiltonian one would expect a splitting of the N@C_{60} single molecule spectral lines with a total distance of $2a$ between the center of the $M_I = -1$ and the $M_I = +1$ peaks. This can be seen utilizing a second order perturbation approach.

We assume that the Electron-Zeeman interaction (H_{EZ}) represents the unperturbed system's Hamiltonian H_0 whereas Hyperfine (H_{HF}) and Zero-Field (H_{ZF}) interactions are considered to be the perturbation H_1:

$$
\begin{aligned}
H &= H_0 \quad + \; H_1 \\
&= H_{EZ} \; + \; (H_{HF} \; + \; H_{ZF}) \; .
\end{aligned}
$$

This yields the energies of the 12 states of the system including their respective energy-corrections as can be seen in Fig. S8. These corrections give the system's level structure as pictured in Fig. S9. The allowed transitions between these energy-states give the spectral lines shown in Fig. S7. The dashed line indicates the result of a measurement with a zero field interaction $D = 1.52$ MHz.

The line broadening is Fourier limited by the experimental pulse width of 100 ns, giving a line broadening of $1/(\pi \times 100\text{ns}) \approx 3.3\,\text{MHz}$ [10]. This is slightly smaller than what we see in the experiment (≈ 4 MHz), indicating that the weak interaction of the spin-bath with the endofullerene is moderately lowering its T_2^* (leading to an additional Gaussian broadening $\propto 1/T_2^*$) [11, 12].

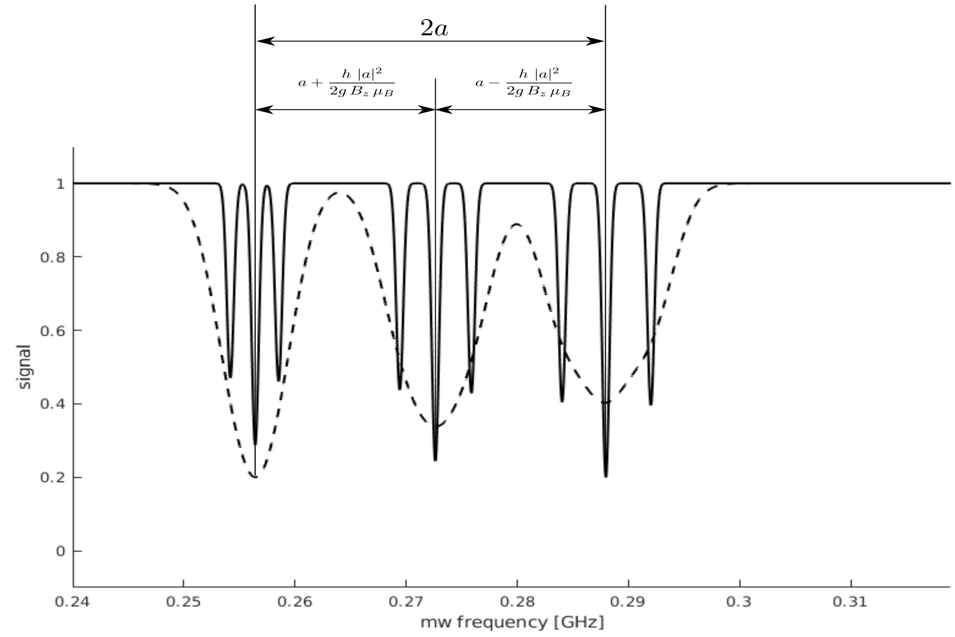

Figure S7: **Spectral simulation for N@C$_{60}$.** N@C$_{60}$ single molecule spectral simulation done with easyspin for a 0.75 MHz line-width (solid line) and a 6 MHz line-width (dashed line) for values of $D = 1.52$ MHz, $a = 15.85$ MHz, $g = 2.00204$ and a z-aligned external magnetic field of $B = 9.697$ mT including 2nd order perturbation calculation of the splitting.

		0. Ord.	1. Ord.		2. Ord.
$M_S = +3/2$	$M_I = +1$	$+\frac{3}{2}\frac{\mu_B}{h}gB_z$	$+\frac{3}{2}a$	$+D$	± 0
$M_S = +3/2$	$M_I = 0$	$+\frac{3}{2}\frac{\mu_B}{h}gB_z$	± 0	$+D$	$+\frac{3}{2}\frac{h}{\mu_B}\frac{a^2}{gB_z}$
$M_S = +3/2$	$M_I = -1$	$+\frac{3}{2}\frac{\mu_B}{h}gB_z$	$-\frac{3}{2}a$	$+D$	$+\frac{3}{2}\frac{h}{\mu_B}\frac{a^2}{gB_z}$
$M_S = +1/2$	$M_I = +1$	$+\frac{1}{2}\frac{\mu_B}{h}gB_z$	$+\frac{1}{2}a$	$-D$	$-\frac{3}{2}\frac{h}{\mu_B}\frac{a^2}{gB_z}$
$M_S = +1/2$	$M_I = 0$	$+\frac{1}{2}\frac{\mu_B}{h}gB_z$	± 0	$-D$	$+\frac{1}{2}\frac{h}{\mu_B}\frac{a^2}{gB_z}$
$M_S = +1/2$	$M_I = -1$	$+\frac{1}{2}\frac{\mu_B}{h}gB_z$	$-\frac{1}{2}a$	$-D$	$+\frac{2h}{\mu_B}\frac{a^2}{gB_z}$
$M_S = -1/2$	$M_I = -1$	$-\frac{1}{2}\frac{\mu_B}{h}gB_z$	$+\frac{1}{2}a$	$-D$	$+\frac{3}{2}\frac{h}{\mu_B}\frac{a^2}{gB_z}$
$M_S = -1/2$	$M_I = 0$	$-\frac{1}{2}\frac{\mu_B}{h}gB_z$	± 0	$-D$	$-\frac{1}{2}\frac{h}{\mu_B}\frac{a^2}{gB_z}$
$M_S = -1/2$	$M_I = +1$	$-\frac{1}{2}\frac{\mu_B}{h}gB_z$	$-\frac{1}{2}a$	$-D$	$-\frac{2h}{\mu_B}\frac{a^2}{gB_z}$
$M_S = -3/2$	$M_I = -1$	$-\frac{3}{2}\frac{\mu_B}{h}gB_z$	$+\frac{3}{2}a$	$+D$	± 0
$M_S = -3/2$	$M_I = 0$	$-\frac{3}{2}\frac{\mu_B}{h}gB_z$	± 0	$+D$	$-\frac{3}{2}\frac{h}{\mu_B}\frac{a^2}{gB_z}$
$M_S = -3/2$	$M_I = +1$	$-\frac{3}{2}\frac{\mu_B}{h}gB_z$	$-\frac{3}{2}a$	$+D$	$-\frac{3}{2}\frac{h}{\mu_B}\frac{a^2}{gB_z}$

Figure S8: **Energy corrections for N@C_{60}.** Energy corrections of a single N@C_{60} molecule calculated from 2nd order perturbation theory.

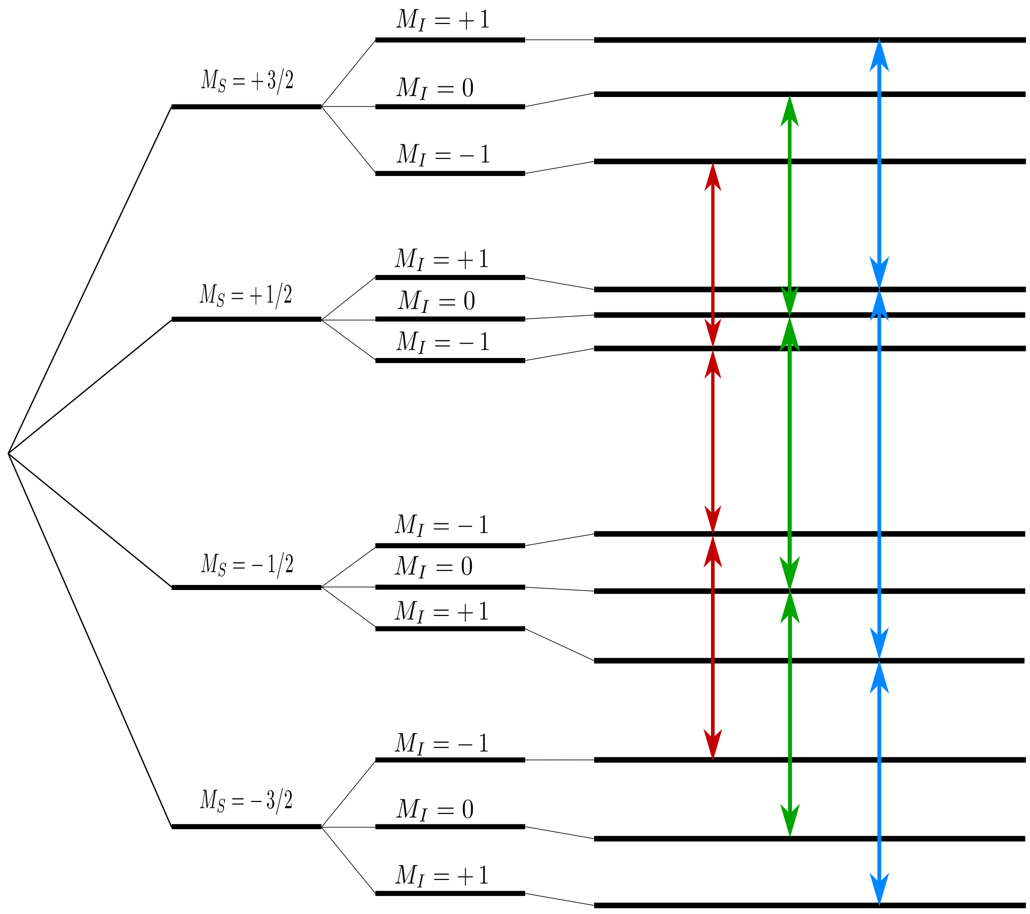

Figure S9: **Corrected energy levels.** Schematic of the level structure of a single N@C_{60} molecule according to the corrections as seen in Figure S8 including the allowed transitions ($\Delta M_S = 0, \pm 1; \Delta M_I = 0$) between the $M_I = -1$ states (red), the $M_I = 0$ states (green) and the $M_I = +1$ states (blue).

2.1 Dependence on zero-field axis

In an ensemble, all axial orientations of the molecule average out. This does not happen at the single spin level. Therefore one might consider an angular dependence when computing the zero-field splitting terms for the single molecule. The angle ϕ between the magnetic field and the orientation of the molecular frame is introduced. The zero-field splitting tensor in the molecular frame is given by:

$$\hat{D}_{mol} = \begin{pmatrix} -\frac{1}{3}D & 0 & 0 \\ 0 & -\frac{1}{3}D & 0 \\ 0 & 0 & \frac{2}{3}D \end{pmatrix} .$$

This now has to be rotated into the lab-frame with a z-aligned magnetic field. For this a rotation around the y-axis over the angle ϕ given by $\hat{R}_y(\phi)\,\hat{D}_{mol}\,\hat{R}_y^T(\phi)$ yields the splitting tensor in the lab-frame

$$\hat{D}_{lab} = \begin{pmatrix} D\left(\cos^2(\phi) - \frac{1}{3}\right) & 0 & \frac{1}{2}D\sin(2\phi) \\ 0 & -\frac{1}{3}D & 0 \\ \frac{1}{2}D\sin(2\phi) & 0 & D\left(\sin^2(\phi) - \frac{1}{3}\right) \end{pmatrix}$$

leading to a zero-field splitting Hamiltonian of the form

$$\frac{\mathcal{H}_{ZF}}{h} = \frac{1}{6}\,D\left(\hat{S}_x^2 + \hat{S}_z^2 - 2\hat{S}_y^2\right) + \frac{1}{2}\,D\left(\hat{S}_z\hat{S}_x + \hat{S}_x\hat{S}_z\right)\sin(2\phi) + \frac{1}{2}\,D\left(\hat{S}_z^2 - \hat{S}_x^2\right)\cos(2\phi)$$

This, as well as an easyspin simulation for different angles shown in Fig. S10, shows that for all angles ϕ the zero-field splitting leads to an almost symmetrical distance between the central transition-peaks (solid lines) for each nuclear quantum number M_I.

Hence, angular dependencies in the zero-field splitting terms do not lead to an effective shift of the peak-positions measurable at a considerable line-width. The total distance between the $M_I = -1$ and the $M_I = +1$ central peaks remains $2a$, which can also be seen from the calculated energy corrections.

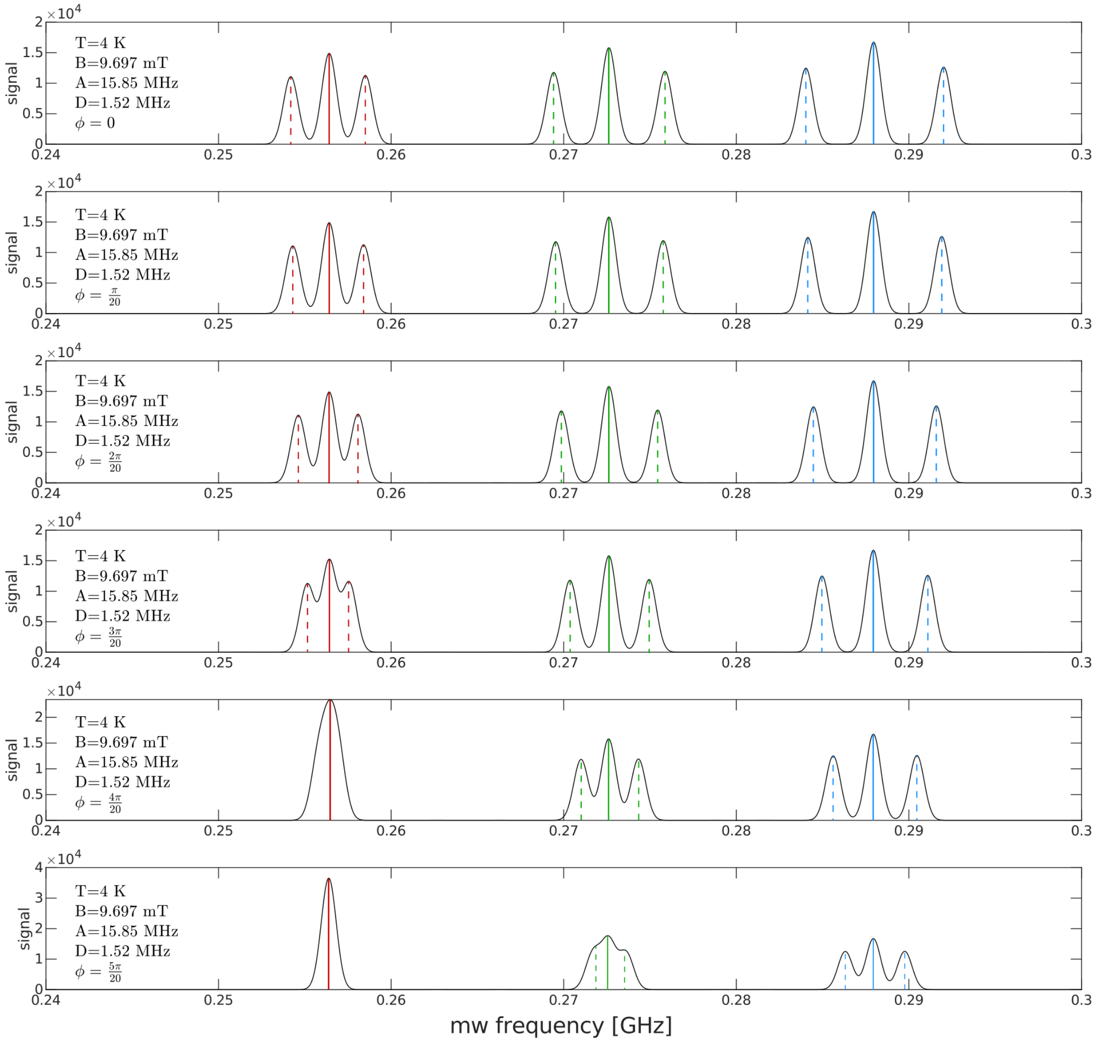

Figure S10: **Dependence on zero-field axis**. Angle dependent zero-field splitting due to an angle ϕ between the z-aligned external magnetic field with $B = 9.697$ mT in the laboratory frame and the molecule's zero-field splitting tensors own z-axis at $D = 1.52$ MHz for a temperature of $T = 4$ K including hyper-fine splitting at $a = 15.85$ MHz with a g-factor of $g(N@C_{60}) = 2.00204$

2.2 Including anisotropic hyperfine interaction

Up to this point the total width of the spectrum is given by $2a$. Ensemble EPR measures $2a$ = 32 MHz for N@C_{60}. However, our single spin EPR results measure $2a$ = 38 MHz. Admitting the possibility of a strong deformation of the C_{60} cage we can consider an anisotropic hyperfine interaction. The anisotropy parameter Δ is introduced so that the interaction tensor $\hat{A}$ becomes

$$\hat{A} = \begin{pmatrix} A_x & 0 & 0 \\ 0 & A_y & 0 \\ 0 & 0 & A_z \end{pmatrix} = \begin{pmatrix} a - \frac{\Delta}{2} & 0 & 0 \\ 0 & a - \frac{\Delta}{2} & 0 \\ 0 & 0 & a + \Delta \end{pmatrix} .$$

The hyperfine interaction Hamiltonian thus reads

$$\begin{aligned} \frac{\mathcal{H}_{\mathrm{HF}}}{h} &= \hat{S}^T \hat{A} \hat{I} \; = \; A_x \hat{S}_x \hat{I}_x + A_y \hat{S}_y \hat{I}_y + A_z \hat{S}_z \hat{I}_z \\ &= \left(a - \frac{\Delta}{2}\right) \left(\hat{S}_x \hat{I}_x + \hat{S}_y \hat{I}_y\right) + (a + \Delta) \hat{S}_z \hat{I}_z \; . \end{aligned}$$

Due to this anisotropy the total distance between the $M_I = -1$ and the $M_I = +1$ central peaks now becomes $2a + 2\Delta$ as can be seen in Fig. S11. This result can also be obtained from the second order perturbation calculation when using the above stated hyperfine Hamiltonian.

When also including an angular dependence in the hyperfine interaction (which should be the same one as for the zero field interaction) the total splitting distance can be found to be $2a + \frac{1}{2}\Delta + \frac{3}{2}\Delta \cos(2\phi)$.

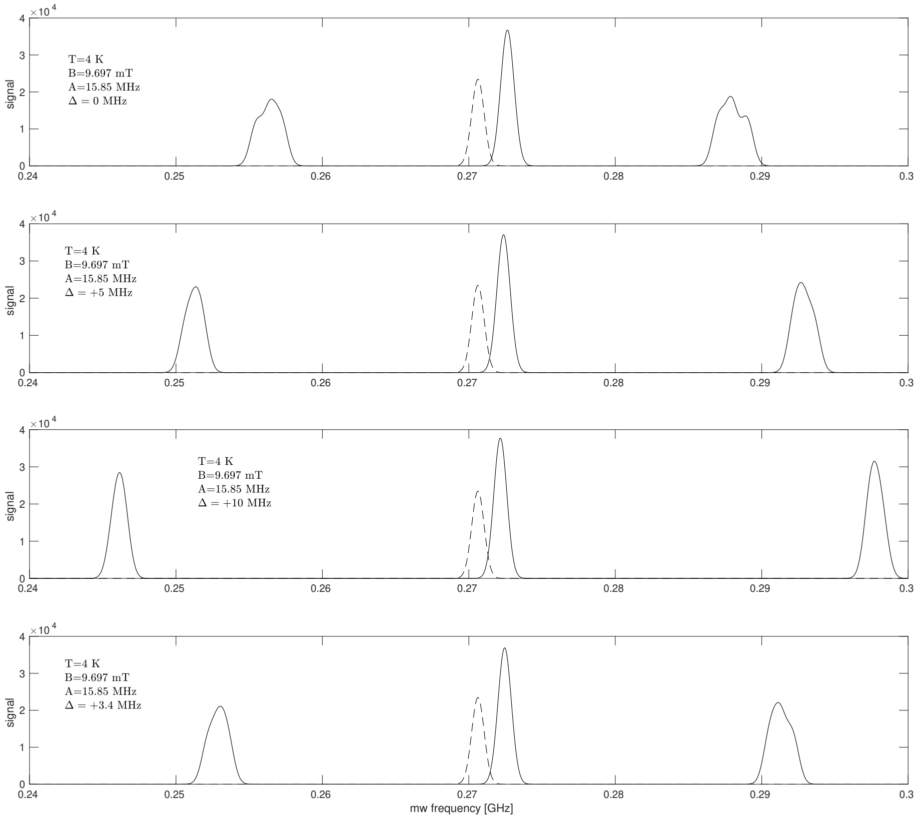

Figure S11: **Anisotropic hyperfine interaction.** Single N@C$_{60}$ molecule spectra for different anisotropy Δ in the hyperfine splitting with $a = 15.85$ MHz without zero-field splitting at a field strength of $B = 9.697$ mT (solid line) and a single C$_{60}$ spectrum (dashed line) with a linewidth of 1 MHz at $g(N@C_{60}) = 2.00204$ and $g(C_{60}) = 1.994$

2.3 Enhanced isotropic hyperfine and axial zero-field interaction

Figure S12 shows a spectral simulation of a single N@C$_{60}$ molecule (brown) with a g-factor of $g_1 = 2.00204$ and a additional spin-1/2 bath (purple) with a g-factor of $g_2 = 2.03$, which may originate from drop coating the N@C$_{60}$ solution. The g-factor of this second spin species, however, strongly differs from the previously considered C$_{60}$ radical. The blue line shows the combined spectrum. Considering a hyperfine constant of $A = 19$ MHz for the spin-3/2 species instead of the usual 15.85 MHz for N@C$_{60}$, the combined spectrum fits the measured data quite nicely even without an anisotropic hyperfine interaction ($\Delta A = 0$) or including an additional angular dependence in the zero-field splitting interaction ($\phi = 0$).

The strong deviation of the N@C$_{60}$ hyperfine parameter could result from the hyperfine constant, possibly being quite different when the molecule is located on the diamond surface as compared to its typical values inside the bulk. This change would imply a decrease of the fullerene cage cavity. Considering all the information gathered from these experiments it however remains unclear

how this specific decrease is caused, and requires follow up experiments to better understand the chemical effects of the diamond surface.

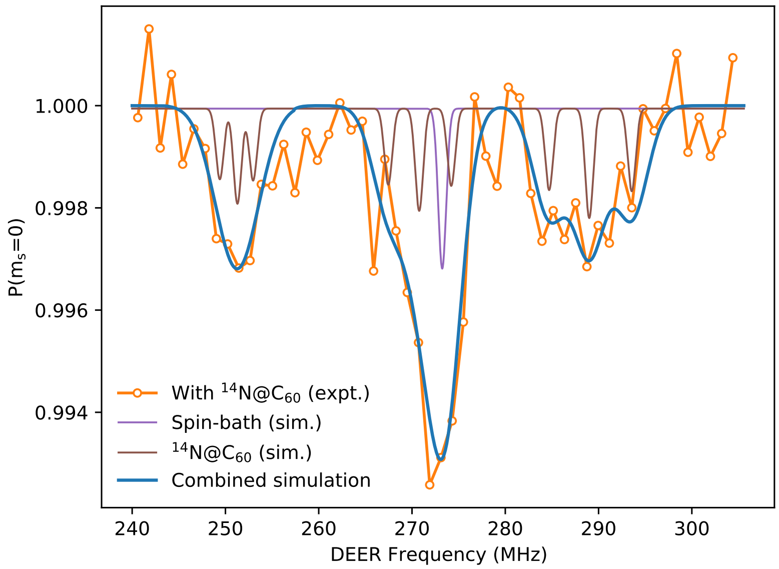

Figure S12: **Enhanced isotropic hyperfine and zero-field interaction.** Comparison of measured (orange) and simulated spectra. The single N@C$_{60}$ is shown with 1 MHz line-width (brown) and 4 MHz line-width (blue) at $T = 4$ K with an isotropic hyperfine splitting $a = 19$ MHz, axial zero-field splitting $D = 1.52$ MHz and a z-aligned magnetic field with $B = 9.697$ mT. The additional S=1/2 bath has an axial g-factor $g = 2.03$.

2.4 Alternative interpretation of peak intensity: Non-thermal spin distributions.

The splitting of hyperfine components in a typical ensemble X-band EPR experiment (9 - 10 GHz) is typically symmetric and can hence only explain the increase of the total spectrum width, not the asymmetry. In the low-field situation here (≈260 MHz), the spectrum may become slightly asymmetric due to a combination of the zfi and second-order hfi terms. The maximum expected downshift would however amount to $A^2/2g\mu_B B_z \approx 0.5$ MHz under the present experimental conditions. The downshift of the lowest-energy transition can be rationalized by considering a possible non-thermal population that favors the minimal angular momentum state of N@C$_{60}$, i.e. $|m_S, m_I\rangle = |{-3/2}, {-1}\rangle$. This would favor the $m_S = {-3/2}$ to $m_S = -1/2$ transition for $m_I = -1$ and hence explain the downshift. A possible driving mechanism could be the frequent inter-system-crossing events of the NV center itself, which happen in the laser polarization intervals whenever the (electronically excited) NV $|{-1}\rangle$ state crosses over to the singlet manifold. This acts as a "drain" for angular momentum in the environment. A detailed picture of the exact sequence of intermediate steps leading to a polarization of the N@C$_{60}$ molecule and related quantitative analysis is beyond the scope of this work. We note that the population redistribution may occur on a long time scale since the spin relaxation times of endofullerenes at 4.7 K are on the order of seconds (T_{1e}) and even hours (T_{1n}) [13]. Each iteration of the measurement sequence is on the order of 5 μs, over time there may well be a detectable accumulation of tiny imbalances of the coupling to the environment. This is shown in Figure S13.-

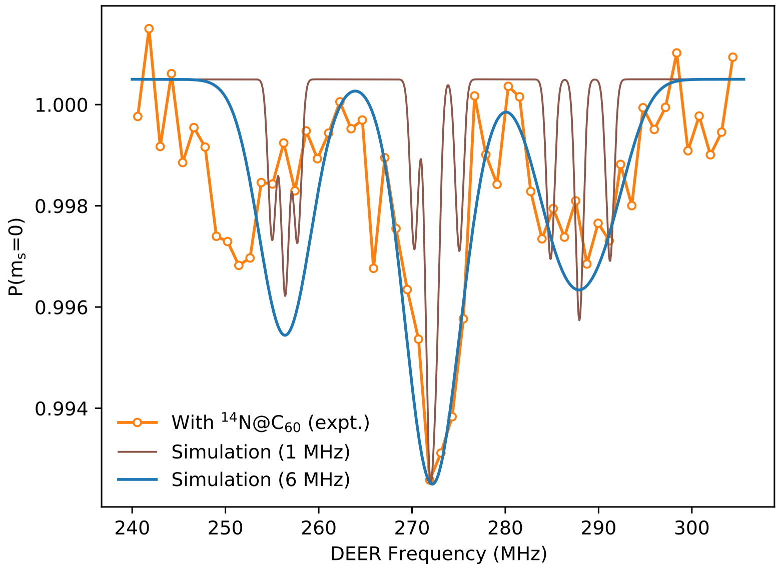

Figure S13: **Non-thermal spin distributions.** Simulated spectra for a $1\,\mathrm{MHz}$ line-width (brown) and a 6 MHz line-width (blue) at temperatures of T = 4 K, hyperfine splitting a = 15.85 MHz, zero-field splitting D = 1.52 MHz and molecular frame rotation of $\phi = 25°$ at a z-aligned magnetic field with $B = 9.697$ mT. Experimental data shown with orange circles.

3 Scalable endofullerene quantum structures

In the main text, we have shown the ability of the single quantum sensor to interact and read out the spins in a single endofullerene molecule. The measured lifetimes and the coupling strengths among various electron and nuclear spins (see Table S14b) clearly indicate a promising quantum system where information could be exchanged among the neighboring electron spins that are controlled by the local hyperfine couplings. Given the long lifetime of the nuclear spins, one could use them as quantum bits and the coupled electron spin chain as a bus mediating their interactions. While such possibilities have been studied earlier in [13], here we would like to focus on a key ingredient i.e., initialization of the quantum systems. Each fullerene molecule represents a 12-level system with a four-level electron spin $S = 3/2$ and a three-level nuclear spin ($I = 1$). Using well-controlled microwave and RF fields, the S spin can be tuned in resonance with the NV electron spin or its intrinsic nuclear spin. Using this, we show in Fig. S14a how the optical polarization of the NV spin can be transferred first to the S spin, which then is finally transferred to the nuclear spin. The polarization transfer in each step is achieved through Hartman-Hahn like transfer employed earlier in Ref. [14] to polarize external spins. In Fig. S14a we show the polarization of the S spin for a fixed m_I, and then repeating the same for all the other m_I transitions we have a fully polarized S spin. The cooling step for a given m_I takes $\sim 30 \times 20$ μs, and for all three nuclear spin states, the total cooling time of the S spin is ~ 3 μs which is quite shorter than its T_1. This polarization can then be transferred to the nuclear spin much more efficiently as they are strongly coupled. We would also like to note that the ultra-strong coupling between the neighboring S spins (50 MHz) does not allow the polarization to the transferred during the $NV - S$ polarization step, allowing to localize the spin polarization only on the endofullerene that is being polarized.

With the possibility to polarize the electron and nuclear spins, and have them transferred throughout the chain to other endofullerene spins, the next steps would be to realize a well-initialized quantum register and observe the dynamics of polarization flow through scalable geometries. Furthermore using multiple NV spins, we should be able to show the fullerene chain mediated correlations between them [8].

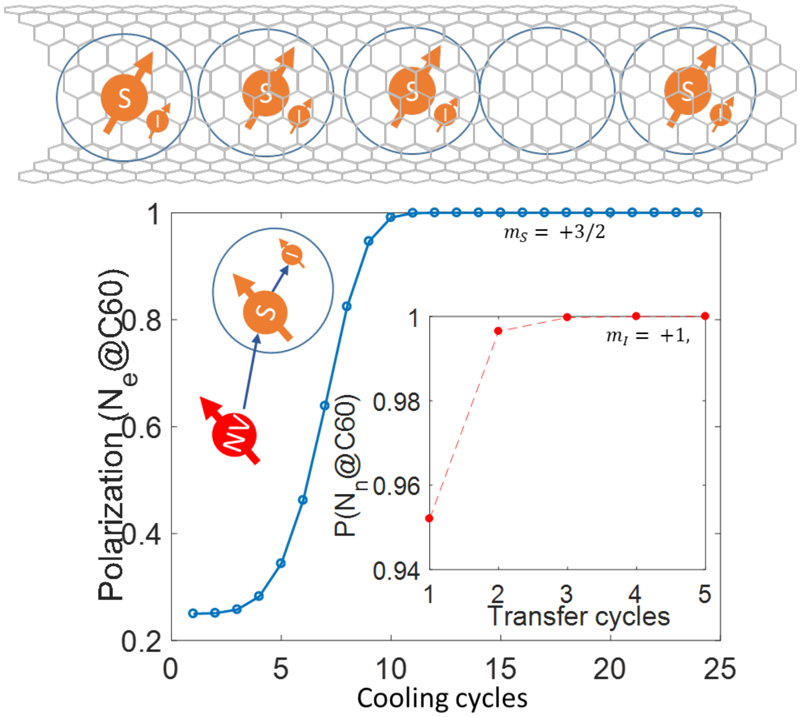

a

	N@C$_{60}$ spin coherence	
	T$_2$	T$_1$
Electronic	200 μs [15]	1 s [16]
Nuclear	130 ms [17]	10^7 s [18]
N@C$_{60}$ hfcc	$A = 15.8$ MHz	[1]
N@C$_{60}$-N@C$_{60}$ coupling	$J \approx 52$ MHz$/(r/\mathrm{nm})^3$	[19]
NV-N@C$_{60}$ coupling (exp.)	0.29 MHz	

b

Figure S14: **Scalable endofullerene quantum structures.** **a**, (Top) Schematic representation of a scalable structure formed using carbon nanotubes to encapsulate the endofullerene molecules. (Bottom) Numerically simulated polarization transfer from a single NV center to a single N@C$_{60}$ electron spin for a given hyperfine transition. The right inset depicts the further transfer of electron polarization to nuclear spin. This polarization scheme is pictorially depicted in the inset (left). For this simulation we have used polarization transfer rates from NV to the electron spin $\tau_{NV-e} = \pi/4J$ and $\tau_{e-n} = \pi/4A$. **b**, Quantitative values of physical parameters such as spin relaxation times and spin-spin coupling used in simulation. hfcc is the N@C$_{60}$ hyperfine coupling constant.

References

[1] Weidinger, A., Waiblinger, M., Pietzak, B. & Almeida Murphy, T. Atomic nitrogen in C 60 :N@C 60. *Applied Physics A: Materials Science & Processing* **66**, 287–292 (1998). URL http://link.springer.com/10.1007/s003390050668.

[2] Scrivens, W. A., Bedworth, P. V. & Tour, J. M. Purification of gram quantities of C60. A new inexpensive and facile method. *Journal of the American Chemical Society* **114**, 7917–7919 (1992). URL https://doi.org/10.1021/ja00046a051.

[3] Momenzadeh, S. A. *et al.* Nanoengineered Diamond Waveguide as a Robust Bright Platform for Nanomagnetometry Using Shallow Nitrogen Vacancy Centers. *Nano Letters* **15**, 165–169 (2015). URL http://pubs.acs.org/doi/10.1021/nl503326t.

[4] Kroto, H. W., Heath, J. R., O'Brien, S. C., Curl, R. F. & Smalley, R. E. C_{60}: Buckminsterfullerene. *Nature* **318**, 162–163 (1985). URL https://doi.org/10.1038/318162a0.

[5] de Lange, G. *et al.* Controlling the quantum dynamics of a mesoscopic spin bath in diamond. *Scientific Reports* **2**, 382 (2012). URL https://www.nature.com/articles/srep00382. Number: 1 Publisher: Nature Publishing Group.

[6] Xing, J., Chang, Y.-C., Wang, N., Liu, G.-Q. & Pan, X.-Y. Electron Spin Decoherence of Nitrogen-Vacancy Center Coupled to Multiple Spin Baths. *Chinese Physics Letters* **33**, 107601 (2016). URL https://doi.org/10.1088%2F0256-307x%2F33%2F10%2F107601. Publisher: IOP Publishing.

[7] Shi, F. *et al.* Single-protein spin resonance spectroscopy under ambient conditions. *Science* **347**, 1135–1138 (2015). URL http://www.sciencemag.org/cgi/doi/10.1126/science.aaa2253.

[8] Schlipf, L. *et al.* A molecular quantum spin network controlled by a single qubit. *Science Advances* **3**, e1701116 (2017). URL http://advances.sciencemag.org/lookup/doi/10.1126/sciadv.1701116.

[9] Schaefer-Nolte, E., Reinhard, F., Ternes, M., Wrachtrup, J. & Kern, K. A diamond-based scanning probe spin sensor operating at low temperature in ultra-high vacuum. *Review of Scientific Instruments* **85**, 013701 (2014). URL http://aip.scitation.org/doi/10.1063/1.4858835.

[10] Vitanov, N. V. *et al.* Power broadening revisited: theory and experiment. *Optics Communications* **199**, 117–126 (2001). URL http://www.sciencedirect.com/science/article/pii/S003040180101495X.

[11] Dréau, A. *et al.* Avoiding power broadening in optically detected magnetic resonance of single NV defects for enhanced dc magnetic field sensitivity. *Physical Review B* **84**, 195204 (2011). URL https://link.aps.org/doi/10.1103/PhysRevB.84.195204. Publisher: American Physical Society.

[12] Kong, F. *et al.* Kilohertz electron paramagnetic resonance spectroscopy of single nitrogen centers at zero magnetic field. *Science Advances* **6**, eaaz8244 (2020). URL https://advances.sciencemag.org/content/6/22/eaaz8244. Publisher: American Association for the Advancement of Science Section: Research Article.

[13] Harneit, W. Spin Quantum Computing with Endohedral Fullerenes. In *Endohedral Fullerenes: Electron Transfer and Spin*, Nanostructure Science and Technology, 297–324 (Springer, Cham, 2017). URL https://link.springer.com/chapter/10.1007/978-3-319-47049-8_14.

[14] Shagieva, F. *et al.* Microwave-Assisted Cross-Polarization of Nuclear Spin Ensembles from Optically Pumped Nitrogen-Vacancy Centers in Diamond. *Nano Letters* **18**, 3731–3737 (2018). URL http://pubs.acs.org/doi/10.1021/acs.nanolett.8b00925.

[15] Naydenov, B. Encapsulation of Endohedral Fullerenes for Quantum Computing (2006). URL https://refubium.fu-berlin.de/handle/fub188/12223.

[16] Meyer, C. Endohedral Fullerenes for Quantum Computing (2003). URL https://refubium.fu-berlin.de/handle/fub188/1646.

[17] Brown, R. M. *et al.* Coherent State Transfer between an Electron and Nuclear Spin in $ˆ{\mathbf{15}}\mathbf{N}\mathbf{@}{\mathbf{C}}_{\mathbf{60}}$. *Physical Review Letters* **106**, 110504 (2011). URL https://link.aps.org/doi/10.1103/PhysRevLett.106.110504.

[18] Morley, G. W. *et al.* Efficient Dynamic Nuclear Polarization at High Magnetic Fields. *Physical Review Letters* **98**, 220501 (2007). URL https://link.aps.org/doi/10.1103/PhysRevLett.98.220501.

[19] Harneit, W. Fullerene-based electron-spin quantum computer. *Physical Review A* **65** (2002). URL https://link.aps.org/doi/10.1103/PhysRevA.65.032322.
